# From literal meaning to veracity in two hundred milliseconds

**DOI:** 10.3389/fnhum.2014.00040

**Published:** 2014-02-04

**Authors:** Clara D. Martin, Xavier Garcia, Audrey Breton, Guillaume Thierry, Albert Costa

**Affiliations:** ^1^Basque Center on Cognition, Brain and LanguageSan Sebastian, Spain; ^2^IKERBASQUEBilbao, Spain; ^3^Department of Technologies and Communication, University Pompeu FabraBarcelona, Spain; ^4^Institut des Sciences Cognitives, University of Lyon – CNRSLyon, France; ^5^Psychology Department, Bangor UniversityBangor, UK; ^6^Institució Catalana de Recerca i Estudis AvançatsBarcelona, Spain

**Keywords:** semantic integration, world knowledge integration, ERPs, N400, P2

## Abstract

Do the integration of semantic information and that of world knowledge occur simultaneously or in sequence during sentence processing? To address this question, we investigated event-related brain potentials elicited by the critical word of English sentences in three conditions: (1) correct; (2) semantic violation; (3) world knowledge violation (semantically correct but factually incorrect). Critically, we opted for low constraint sentence contexts (i.e., whilst being semantically congruent with the sentence context, critical words had low cloze probability). The processing of semantic violations differed from that of correct sentences as early as the P2 time-window. In the N400 time-window, the processing of semantic and world knowledge violations both differed significantly from that of correct sentences and differed significantly from one another. Overall, our results show that the brain needs approximately 200 ms more to detect a world knowledge violation than a semantic one.

## Introduction

Most people know that the capital of France is Paris, not Barcelona, or that Big Ben can be found in London, not Madrid. Factual information about the world stored in long-term memory—i.e., world knowledge—is constantly retrieved when processing language to make sense of spoken or written content. Comprehenders do not only rely on definitional knowledge of words and expressions (i.e., literal semantics), they also form expectancies from and confront semantic content against world knowledge, which enables them to evaluate information plausibility, modify existing representations, and form opinions. It is this information -not conveyed literally- which leads to perceive the following statement “I am going to Madrid next week, so I will visit Big Ben” as a lie, a confusion, or perhaps a joke. Understanding the cognitive mechanisms underlying language comprehension therefore requires a detailed understanding of the way in which literal semantics and world knowledge are accessed and integrated.

Here, we investigated whether readers retrieve and integrate literal semantic and world knowledge information simultaneously or in sequence during sentence comprehension.

This question is important because two mainstream theories predict opposite results: According to the “dissociation theory” (Forster, 1979), literal semantic integration precedes world knowledge integration, whilst “simultaneous theory” (Jackendoff, 2002) argues in favor of simultaneous integration since the meaning of a word can be fully established only by invoking world knowledge. By literal meaning (or semantics), we refer to definitional knowledge of words, sentences, expressions as it is constrained by the language in use. Sentences violating literal meaning are sentences somehow ill-formed, which violate semantic constraints having to do with the possibilities of combining words in sentences. For instance, “He got married with a stone” is considered a semantic violation because the sentence has no literal meaningfulness (although it may have a metaphorical one), because “getting married with” requires an animate argument. Whereas, on the one hand, grammar constrains the range of legal utterances, on the other hand, humans never produce random legal utterances because language is used to communicate about the world, and the organization of the world therefore also constrains language use. In that sense, some utterances can be semantically correct but contextually inappropriate, and only subsets of semantically correct utterances make sense when invoking world knowledge. By world knowledge we refer to factual information about the world stored in long-term memory and constraining the plausibility of expressions. Sentences violating world knowledge are sentences that describe situations that do not fit our knowledge of a person, a situation, or an event. For instance, the sentence “He spent holidays on Mars” violates common knowledge because it is currently impossible to travel to and/or stay on Mars. Another example is “Barack Obama is the president of France.” This exemplifies a world knowledge violation since, despite the coherent structure and interpretability of the sentence, it is factually incorrect. This distinction between semantic acceptability (coherent or not) and truth value (true or false) is the focus of the present study.

To study the time-course of semantic and world knowledge integration, we recorded event-related brain potentials (ERPs) in English readers presented with sentences containing either literal semantic or world knowledge violations. Hagoort et al. (2004) previously compared ERPs elicited by critical words that completed (1) correct and true sentences, (2) sentences with semantic violations, and (3) sentences with world knowledge violations (false sentences). They observed that the N400 component associated with literal semantic and world knowledge violations had a similar latency, suggesting that “while reading a sentence, the brain retrieves and integrates word's meaning and world knowledge at the same time” (Hagoort et al., 2004). In the present study, we also investigated literal semantic and world knowledge violations but in a slightly different way to Hagoort et al. The motivation for experimental variations is explained in the following paragraphs. We set out (1) to analyse ERP data based on individual world knowledge rather than common and general knowledge, (2) to use sentences with low constraint contexts, and (3) to focus on early semantically driven differences occurring before the window of the classical N400 effect (e.g., Kutas and Federmeier, 2000, 2011).

### ERP data analysis based on individual world knowledge

One of the problems inherent to the study of world knowledge is that each individual has a different and unique knowledge of the world. To address this issue we analyzed ERP data taking into account participant's knowledge as tested by our experimental sentences. After the ERP recording session, each participant was presented again with the experimental material and asked to make true/false/don't know judgments on each sentence. This information was then used to select the trials included in the averaging to generate three ERP: (a) true, (b) false, and (c) don't know.

There were two main reasons for taking into account participant's individual knowledge. First, as in Hagoort et al. (2004)'s study, some sentences reflected common knowledge (e.g., “what children do or not before the age of 8”) and other reflected general knowledge (e.g., “who were the Beatles”; cf. Table 1). General knowledge is prone to inter-individual variability since participants do not systematically share the same knowledge^1^. In order to remove noise from the data, we took into account individual knowledge in such a way that true sentences were all actually true and false sentences were false for each participant. Second, this gave us the opportunity to explore ERPs elicited by sentences for which the participants had no correct representation (“don't know” condition). Such data analysis based on individual world knowledge is new (Hagoort et al., 2004; Hald et al., 2006, 2007) and should increase the signal-to-noise ratio of the experiment, allowing us to observe ERP modulations by world knowledge violation in more details than more classical approaches.

**Table 1:** **Examples of sentences used as experimental material**.

**Sentences**	**Conditions**		
	**Correct**	**WK violation**	**Semantic violation**
Before the age of eight, children start to… and to write.	Read	Smoke	Bark
People go to parks when they want to… and have a walk.	Rest	Buy	Bite
When it is rainy, people cannot… as though it's sunny.	Tan	Speak	Meow
Mines are… and dangerous.	Dark	Crowded	Happy
During summer, many women wear… and dresses.	Sandals	Boots	Carrots
During underwater diving sessions it is common to see… and starfish.	Jellyfish	Eagles	Smells
The Beatles were… in the 60's.	Popstars	Lawyers	Horses
The Egyptian pyramids are very… buildings.	Old	Small	Savory
Santa Claus is very… and famous.	Friendly	Young	Bumpy
The football player Maradona was a… in the Argentinean team.	Forward	Goalkeeper	Dress
Everest is a… and tall mountain.	Snowy	Tropical	Studious
Pope Benedict XVI is… and lives in the Vatican.	GERMAN	Asian	Pollinated

### Sentence context influence on semantic processing

Sentence context has a major impact on word processing and word-sentence integration processes (see for instance Fischler and Bloom, 1979; Stanovich and West, 1979; Kleiman, 1980). The level of constraint imposed by the context determines the extent to which upcoming words can be anticipated. Previous studies have shown that, when sentence context is highly constrained, any critical word different from the anticipated one elicits greater N400 ERP amplitude. For instance, when participants read the sentence “The day was breezy so the boy went outside to fly …,” the presentation of “an airplane” increases N400 amplitude relative to the expected “a kite,” even though this ending is acceptable both in terms of literal semantics and world knowledge (Federmeier and Kutas, 1999a,b; Federmeier et al., 2002; DeLong et al., 2005).

Moreover, it is already established that both literal semantic and world knowledge violations elicit N400 modulation (Kutas and Federmeier, 2000; Hagoort et al., 2004; Hald et al., 2007). Thus, we know that “literal and factual knowledge integration” and “anticipation” influence word processing in the same time-window. In order to reduce the potential contribution of anticipatory processes, we only used low constraint sentences in the present study (i.e., sentences in which upcoming words could not be anticipated). Some sentences used in Hagoort et al. (2004)' study were highly constrained, such that there was only one critical word that could complete the sentence (e.g., “The fall of the Berlin Wall reunited *Germany*”). Thus, in this particular case, any critical word that is not the “only possible completion” is likely to be processed as invalid (i.e., violating the expectancy), and will elicit a larger N400. It is then possible that this large N400 due to expectancy violation could mask more subtle N400 modulations dependent on the type of violation, e.g., semantic vs. world knowledge. Thus, to avoid confusion between the effects of anticipation and those elicited by semantic and world knowledge violations, we chose to use low constraint sentences.

The use of low constraint sentences was the main difference between Hagoort et al. (2004)'s and our study. Hagoort et al. showed that world knowledge and semantic violations are processed in the same way until 480 ms after stimulus onset, when both violations primarily violate a strong lexical expectation based on the sentence context (in some of the trials at least). In the present experiment, we studied similar types of violations within low constraint contexts. We thus investigated how violations are processed in a context where lexical expectation is not the main effect driving semantic integration. Previous research has shown that the influence of contextual integration on sentence processing is highly dependent on stimulus variance and probability of occurrence (Sereno and Rayner, 2003; Penolazzi et al., 2007). Our main hypothesis was that literal semantic integration would precede world knowledge integration (Forster, 1979) in the case of sentences with low constraint contexts. In other words, we tested the hypothesis that previous reports of similar time-course of semantic integration for the two violation types were an artifact caused by high-level of lexical expectancy. For examples, the words “*Vietnam*” and “*gravity*,” despite representing different types of semantic violation are both markedly unexpected vis-à-vis the highly expected ending “Germany,” possibly making the violation more similar.

### Early ERP modulations during word integration

Thirty years of research have strongly established the modulation of the N400 component by semantic integration difficulty during sentence comprehension (Kutas and Hillyard, 1980, 1984; Kutas and Federmeier, 2000, 2011). However, the existence of semantically dependent modulations beyond 350 ms does not preclude stages of semantic integration occurring in earlier time-windows. In fact, several studies have suggested that semantic processing differences may be detectable as early as 150–200 ms after critical stimulus onset. For instance, Landi and Perfetti (2007) observed an early sensitivity to semantic incongruity at around 150 ms (P2 range) when target words were preceded by semantically unrelated prime words (see also Baccino and Manunta, 2005; Wirth et al., 2008). In a sentence reading task, Penolazzi et al. (2007) observed effect of semantic context integration within 200 ms of critical word onset, well before the N400 time-window. In a recent study, Pinheiro et al. (2010) observed that the P2 component was larger for semantically congruent as compared to incongruent critical words presented at the end of a sentence. Moreover, in several previous studies using low constrained sentences (as used in the present study), the P2 component tended to be larger for correct sentences than for sentences with semantic violations (Federmeier and Kutas, 1999a,b, 2002; Federmeier et al., 2002; Wlotko and Federmeier, 2007). Studies investigating the recognition potential (RP) component (peaking around 250 ms after stimulus onset) have also detected early sensitivity to semantic manipulations (Martín-Loeches et al., 2001). For example, in a sentence reading task with semantic context manipulation, Martín-Loeches et al. (2004) reported that the RP component was larger for contextually congruent as compared to incongruent words. Altogether, these previous studies argue for the existence of semantic understanding and contextual integration influence early during sentence comprehension, that is earlier than the traditional N400 time-window, in the range of the P2 and RP components (200–250 ms after stimulus onset, Martín-Loeches et al., 2004; Landi and Perfetti, 2007; Penolazzi et al., 2007; Pinheiro et al., 2010; Regel et al., 2010; see also Barber and Kutas, 2007; Pulvermüller, 2001; Pulvermüller et al., 2001, 2009). Such early time-window analyses were not reported by Hagoort et al. (2004). Since only one electrode was presented in the article's figure, potential early effects of semantic violation cannot be determined. Thus, focusing on semantic violation effects earlier than the N400 time-window is another important contribution of the present study compared to previous ones.

Since the main goal of the present study was to establish the temporal sequence of events during the integration of literal semantic and world knowledge information, we analyzed violation effects not only in the N400 but also the P2 time range. In the studies revealing early semantic incongruity effects mentioned above, the P2 component was larger for semantically related as compared to semantically unrelated words (in word pairs or sentences; Landi and Perfetti, 2007; Penolazzi et al., 2007; Pinheiro et al., 2010). Thus, in the present study, we hypothesized that the P2 component would be larger for correct words compared to words eliciting semantic violations. If the P2 component was exclusively sensitive to semantic congruency, it should not be modulated by words eliciting world knowledge violations.

Regarding the N400, we expected to observe significant modulations for semantic and world knowledge violations, with a larger effect for literal semantic violations as compared to world knowledge ones, as reported previously by Hagoort et al. (2004).

## Materials and methods

### Participants

Eighteen native English speakers (12 females; mean age = 20.6 years ±3.7) took part in the experiment. All participants gave written consent to take part in the study that was approved by the ethics committee of Bangor University, Wales, UK.

### Task and procedure

Stimuli consisted of three versions of 120 sentences: (1) correct and true sentences such as “In a jewellery store one can buy *bracelets* and rings” (critical word in italics); (2) sentences with world knowledge violations as “In a jewellery store one can buy *croissants* and rings” (semantically correct but false); (3) sentences with semantic violations as “In a jewellery store one can buy *brains* and rings” (see Table 1). Three lists of 120 sentences were created, each of them containing 40 sentences of each condition. Each sentence was used only once per list, in one of the three versions. Each participant was randomly assigned to one list. The 120 sentences were mixed with 120 filler neutral sentences, which were not analyzed. Filler sentences were semantically and syntactically congruent and did not refer to common and general knowledge (e.g., “Peter waited for Ana because he wanted to speak to her”). Sentences were randomly presented for each participant inside a given list.

Importantly, the critical word in correct sentences was neither the only possible candidate nor the most expected candidate to complete the sentence. For instance, the sentence “In a jewellery store one can buy… ” can be completed with the words rings, diamonds, necklaces, pearls, etc. A Cloze probability^2^ rating test was administered to 39 participants who did not participate in the experiment. The critical word of correct sentences had an averaged cloze probability of 8.9% ±9 (range 0–44%) and was, on average, the third most expected word (Average cloze probability of the first and second best completions: 28.0% ± 11 and 13.6% ± 6 respectively). The critical words of sentences with world knowledge violations and semantic violations had an averaged cloze probability of 0.0 and 0.0% respectively. In addition, the critical word was never the last word of the sentence. The critical words were matched across conditions on the following criteria: average length in characters (*p* = 0.90) and syllables (*p* = 0.62), log-word frequency (*p* = 0.17), concreteness (*p* = 0.23), imageability (*p* = 0.20) and word class (equated within each pair; see Table 2 for numerical values). Finally, working memory requirements were balanced between semantic and world knowledge violations: The distance between the violation and the word in the sentence that revealed the violation did not significantly differ between conditions (3.6 ± 1.6 words in the WK violation condition; 3.4 ± 1.5 words in the semantic violation condition; *t*-test: *p* = 0.18).

**Table 2:** **Critical word criteria controlled across conditions**.

	**CS**	**WK**	**SV**
Length in characters	6.4 (2.1)	6.3 (2.1)	6.3 (2.0)
Syllable number	1.8 (0.7)	1.7 (0.8)	1.8 (0.8)
Log- word frequency	1.6 (0.7)	1.6 (0.6)	1.4 (0.5)
Concreteness	510 (115)	472 (110)	507 (108)
Imageability	521 (99)	514 (78)	546 (85)

CS, correct sentences; WK, sentences with world knowledge violations; SV, sentences with semantic violations. Standard deviations are reported into bracket.

Each sentence was presented centrally, one word at a time (200 ms duration and 500 ms stimulus onset asynchrony). Sentences were separated by a fixation cross displayed for 800 ms. The instruction was to read each sentence silently and to answer yes or no to the subsequent comprehension question (when applicable; ¼ of the trials) by pressing Y or N buttons on a response pad. The latter quiz test ensured that participants processed sentence meaning during silent reading.

At the end of the experiment, participants were asked to perform a surprise follow-up test. The 80 true and false sentences were presented on the screen along with a rating scale. Participants had to rate each sentence as true or false by pressing “1” or “2.” They had to press “3” if they did not know if the sentence was true or false and “4” if they could not decide because the sentence was meaningless.

### Electrophysiological recording and data analyses

Electrophysiological data were recorded (Scan 4.3; Neuroscan, Inc., El Paso, TX, USA) in reference to electrode Cz at a rate of 1kHz from 64 Ag/AgCl electrodes placed according to the 10–20 convention. Vertical and horizontal EOG were recorded simultaneously with EEG. Impedances were kept below 5 kOhm. EEG activity was filtered off-line [0.1–30 Hz]. Eye blink artifacts were mathematically corrected using the Gratton and Coles's (1989), implemented in Brain Vision Analyzer 2.0 (Brain Products, München), and any remaining artifacts were manually dismissed. Epochs ranged from −100 to 700 ms, time 0 ms being the onset of the critical word of each sentence. Baseline correction was performed in reference to pre-stimulus activity (from −100 to 0 ms) and individual averages were digitally re-referenced offline to the mean of left and right mastoid signals. P2 and N400 components were analyzed over a subset of 36 electrodes where activity was maximal based on the global field power activity. P2 mean amplitude was measured as the average of the ERP amplitude in the [150–200] ms time-window and N400 mean amplitude was measured as the average of the ERP amplitude in the [350–550] ms time-window, both at 36 electrode sites (Left Frontal scalp: F3, F5, F7, FC1, FC3, FC5; Left Central scalp: C1, C3, C5, CP1, CP3, CP5; Left Parietal: P1, P3, P5, PO3, PO7, PO9; Right Frontal scalp: F4, F6, F8, FC2, FC4, FC6; Right Central scalp: C2, C4, C6, CP2, CP4, CP6; Right Parietal: P2, P4, P6, PO4, PO8, PO10). The channel sub-selection was the same for all subjects and peaks. Mean amplitudes of the P2 and N400 peaks were analyzed using a 3 × 3 × 2 repeated measure analysis of variance (ANOVA). The ANOVA factors were Condition [Correct sentence (CS) vs. World Knowledge violation (WK) vs. Semantic violation (SV)], Region (Frontal vs. Central vs. Parietal) and Hemisphere (Left vs. Right). The onset of significant differences between conditions was measured using ms-by-ms paired *t*-tests for the contrasts of interest (SV vs. CS and WK vs. CS; analyses performed on the subset of 24 frontal and central electrodes used for previous statistical analyses and for which the condition effect was significant). Unstable differences (remaining below *p* = 0.05 for less than 30 ms) were discarded (Rugg et al., 1993).

## Results

### Behavioral results

Accuracy in the quiz test was of 85.6% ± 7.9. In the follow-up test, participants rated 80% ±9 of correct sentences as true (6% ± 5 as false and 14% ± 10 as “Don't know”). They rated 73% ± 11 of WK sentences as false (10% ± 8 as true and 17% ± 10 as “Don't know”). In order to take into account individual world knowledge, four ERP conditions were computed: (1) correct sentences, rated as true in the follow-up test; (2) world knowledge violations (WK), rated as false; (3) “don't know” sentences (DK), corresponding to cases in which participants had insufficient knowledge to make a decision; and (4) semantic violations (SV). Overall, 30% ± 4 of the sentences were considered as correct, 26% ± 4 as WK, 10% ± 6 as DK, and 33% ± 0 as SV. Among the 30% of sentences considered as correct, 89% ± 7 were originally true and 11% ± 7 were false. Among the 26% of sentences considered as world knowledge violations, 93% ± 6 were originally false and 7% ± 6 were true. Among the 10% of sentences of sentences of the Don't know condition, 44% ± 11 were originally true and 56% ± 12 were false.

When each condition was computed taking into account individual knowledge, the critical word of true sentences had an averaged cloze probability of 8.02% ± 1 (range 0–44%). The critical words of sentences with world knowledge violations and semantic violations had an averaged cloze probability of 0.68% ± 0.9 and 0.0% respectively. The critical words of “Don't know” sentences had an averaged cloze probability of 3.55% ± 2. The critical words were still matched across the four conditions on the following criteria: average length in characters (*p* = 0.58) and syllables (*p* = 0.29), log-word frequency (*p* = 0.06), concreteness (*p* = 0.25), imageability (*p* = 0.24) and word class (equated within each pair).

### ERP results

**Figure 1:**
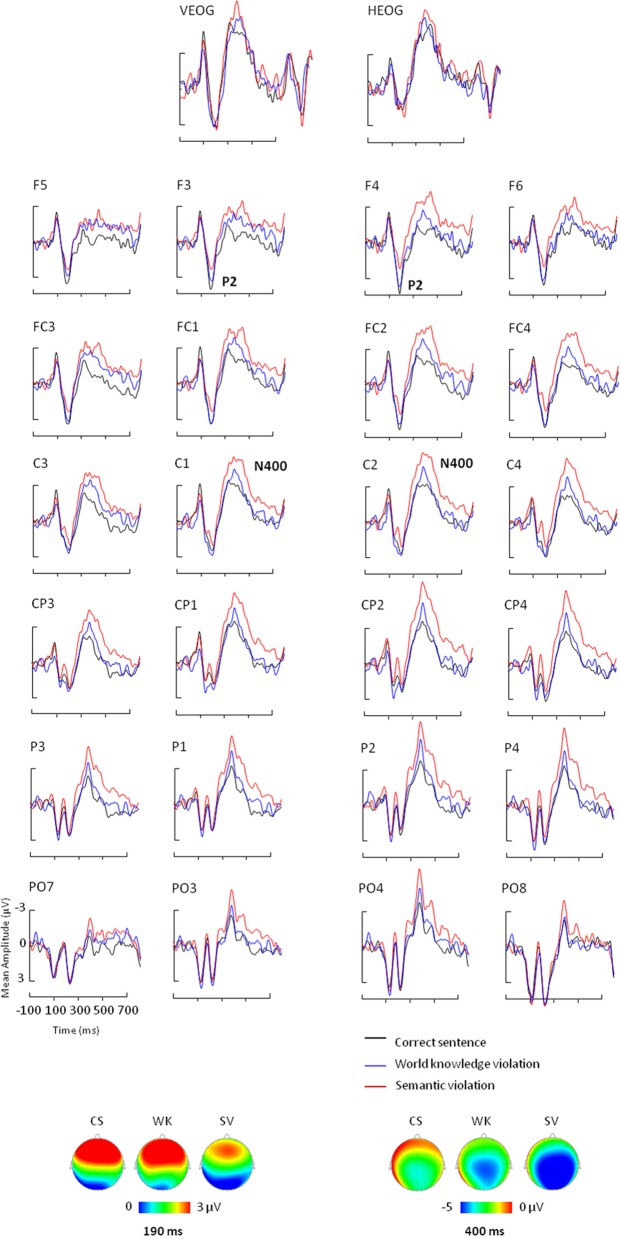
**Event-related potential results for correct sentences (black lines), sentences with world knowledge violations (blue lines), and sentences with semantic violations (red lines)**. ERPs measured in the [–100; 700] ms time-window over VEOG, HEOG, 4 frontal, 4 frontocentral, 4 central, 4 centroparietal, 4 parietal, and 4 parieto-occipital electrodes. Negativity is plotted up. Topographic distribution of the correct condition (CS), the semantic violation condition (SV), and the world knowledge violation condition (WK) at 190 ms (bottom left) and 400 ms (bottom right).

**Figure 2:**
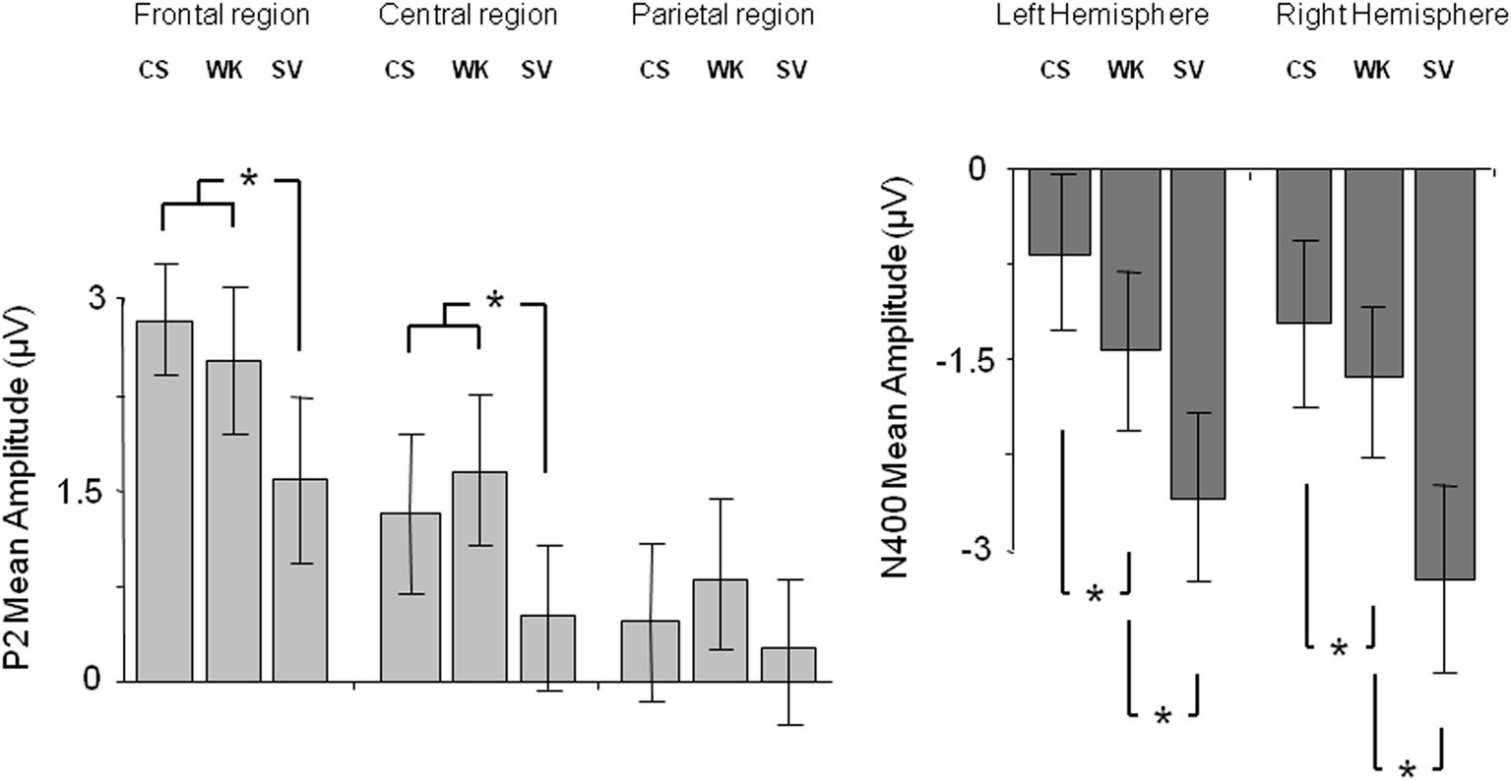
**P2 mean amplitudes over the frontal, central, and parietal regions (left panel) and N400 mean amplitudes over the left and right hemispheres (right panel), for correct sentences (CS), sentences with world knowledge violations (WK), and sentences with semantic violations (SV)**. Stars indicate significant differences between conditions. Error bars depict standard errors.

ERPs for each condition were obtained by averaging individual data taking into account individual knowledge and removing trials with artifacts in the EEG signal. Statistical analyses were performed on average on 34 ± 4 trials for the True condition, 29 ± 5 trials for the False condition, 11 ± 5 trials for the DK condition and 38 ± 5 trials for the SV condition for each participant. Table 3 shows the ANOVA results and *post-hoc* analyses on P2 and N400 mean amplitudes. Figure 1 depicts the ERPs elicited by correct sentences, semantic violations and world knowledge violations. Figure 2 shows ERP mean amplitude values for the same conditions.

### ERP P2 results

The ANOVA performed on P2 mean amplitudes revealed significant effects of condition and region and a significant condition x region interaction (see Table 3A for statistical results). There was no hemispheric effect, no condition × hemisphere interaction, no hemisphere × region interaction and no triple interaction. *Post-hoc* analysis of the condition × region interaction (Bonferroni test; see Table 3B) revealed that P2 differences were due to semantic violations eliciting smaller P2 amplitudes as compared to the world knowledge violations and correct sentences, over frontal and central regions. CS and WK sentences did not differ over any of the two regions. No condition effects were observed over parietal region (see Figure 1 for ERP waves and Figure 2 for mean amplitude values). Thus, P2 mean amplitude was sensitive to semantic violations but not to world knowledge violations.

**Table 3A:**
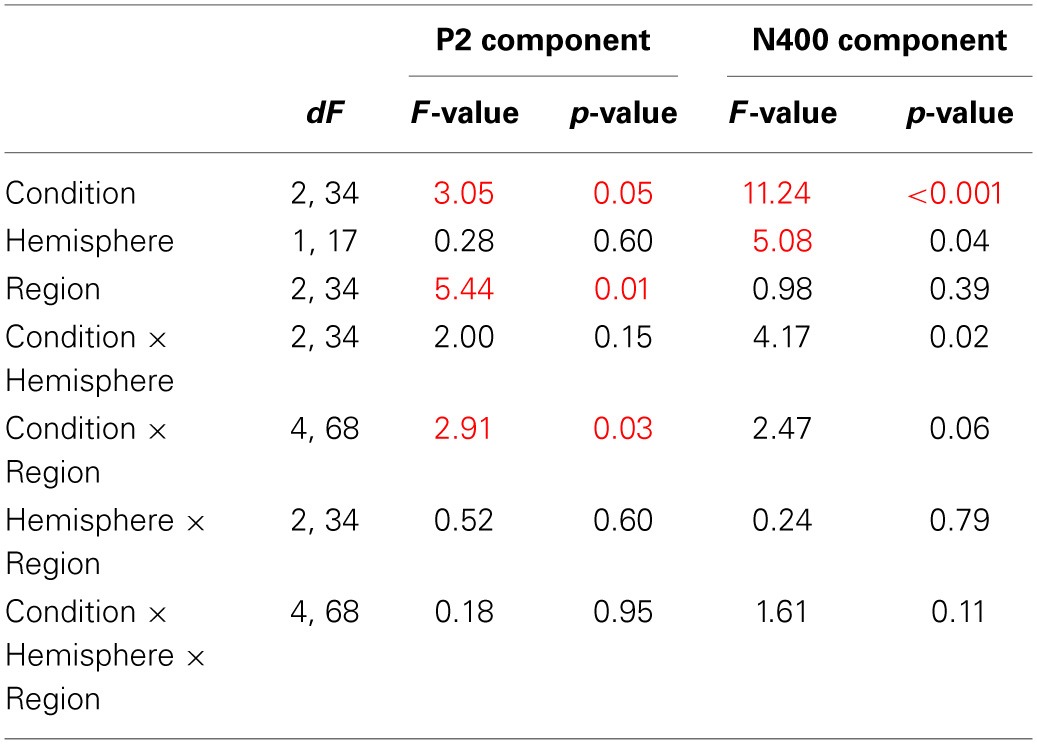
**General ANOVA for CS vs. WK vs. SV comparison**.

CS, correct sentences; WK, world knowledge violations; SV, semantic violations; dF, degree of freedom; Significant effects and interactions are labeled in red.

**Table 3B:**
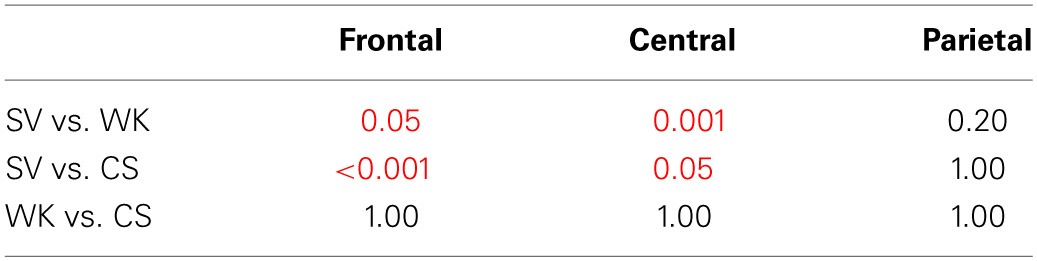
**P2 *Post-hoc* analysis—Bonferroni test of the condition × region interaction**.

### ERP N400 results

The general ANOVA performed on N400 mean amplitudes revealed significant effects of condition and hemisphere and a significant condition x hemisphere interaction (see Table 3A for statistical results). There was no region effect, no condition × region interaction, no hemisphere × region interaction and no triple interaction. *Post-hoc* analysis of the condition × hemisphere interaction (Bonferroni test; see Table 3C) revealed that the three conditions differed from each other over both hemispheres: SV sentences elicited larger N400 mean amplitude than WK sentences and than correct sentences. WK sentences elicited larger N400 mean amplitude than correct sentences. N400 mean amplitude was larger over the right than the left hemisphere in SV sentences and did not vary over hemispheres in WK and correct sentences (see Figures 1, 2). Thus, N400 mean amplitude was sensitive to both semantic and world knowledge violations, being larger for the former condition.

**Table 3C:**
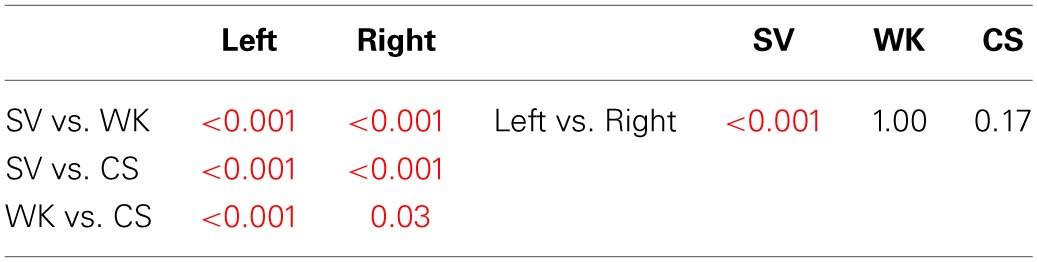
**N400 *Post-hoc* analysis—Bonferroni test of the condition × hemisphere interaction**.

### Ms-by-ms paired *t-test* analysis

**Figure 3:**
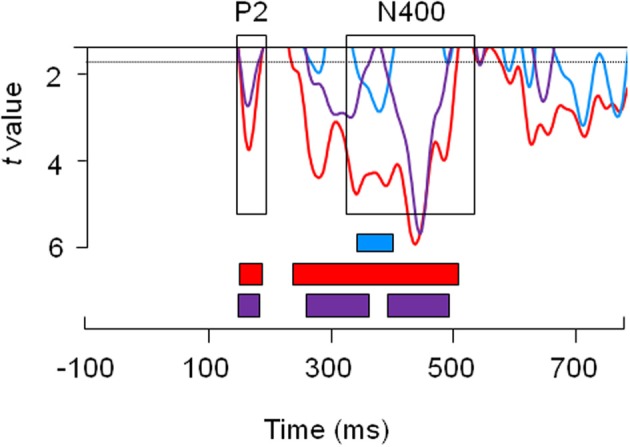
**Paired *t*-test analysis comparing semantic violation (SV) and correct sentence (CS) conditions (red line), comparing world knowledge (WK) and correct sentence (CS) conditions (blue line) and comparing world knowledge (WK) and semantic violation (SV) conditions (purple line)**. *T*-values are plotted for the entire time-window of analysis [(−100; 700) ms, time 0 coinciding with the presentation of the critical word]. The upper horizontal line represents a threshold of 0.1 significance level. The horizontal dotted line represents the 0.05 significance level. The blue square indicates reliable significant differences between WK and CS conditions. The red squares indicate the temporal windows with reliable significant differences between SV and CS conditions. The purple squares indicate the temporal windows with reliable significant differences between WK and SV conditions.

To gain a more fine-grained analysis of these effects, a ms-by-ms paired *t*-test analysis was conducted, in which we compared SV and WK sentences against correct sentences (CS; see Figure 3). That is, we compared the amplitude of brain responses for each of the violation conditions against the control condition every millisecond, i.e., a component-independent analysis. We also compared SV sentences against WK sentences. The first sustained significant differences (remaining below *p* = 0.05 for more than 30 ms) between SV and CS were found at 150 and 240 ms. In contrast, the first sustained significant differences between WK and CS were found only at around 350 ms. WK and SV conditions started to significantly differ at 150 ms and then again at 260 ms.

### Further analyses on P2 and N400 ERP components

**Figure 4:**
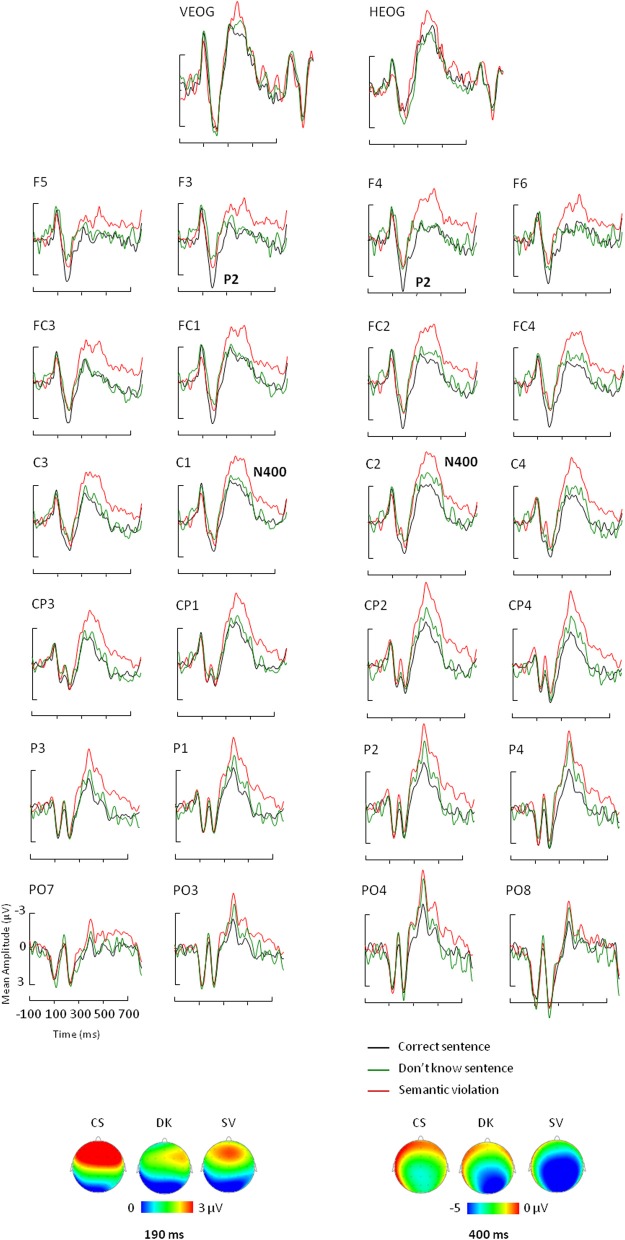
**Event-related potential results for correct sentences (black lines), “Don't know” sentences (green lines) and sentences with semantic violations (red lines)**. ERPs measured in the [–100; 700] ms time-window over VEOG, HEOG, 4 frontal, 4 frontocentral, 4 central, 4 centroparietal, 4 parietal, and 4 parieto-occipital electrodes. Negativity is plotted up. Topographic distribution of the correct condition (CS), the semantic violation condition (SV), and the “Don't know” condition (DK) at 190 ms (bottom left) and 400 ms (bottom right).

**Figure 5:**
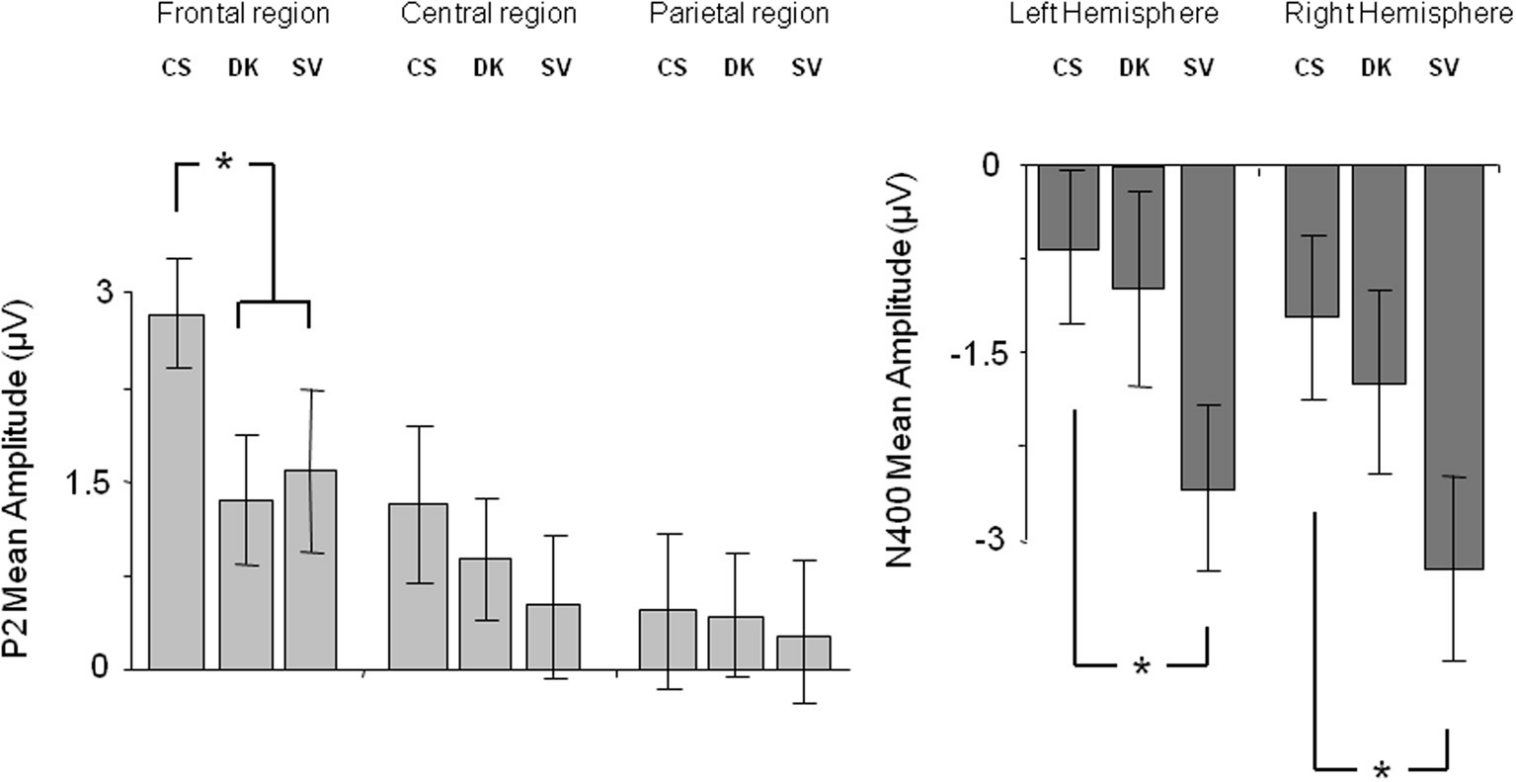
**P2 mean amplitudes over the frontal, central, and parietal regions (left panel) and N400 mean amplitudes over the left and right hemispheres (right panel), for correct sentences (CS), “Don't know” sentences (DK) and sentences with semantic violations (SV)**. Stars indicate significant differences between conditions. Error bars depict standard errors.

A potential caveat when interpreting differences between SV and correct sentences in the P2 time-window is the fact that they might stem from amplitude shifts appearing later in the N400 time-window. In other words, smaller P2 mean amplitude for SV as compared to correct sentences might be a byproduct of the larger N400 mean amplitude elicited by SV critical words rather than diverging cognitive processes starting between 150–200 ms. On the other hand, differences between violation conditions and baseline condition in the N400 time-window might also be explained as a carry-over effect of the differences appearing in the P2 time-window. To address this issue, we performed three additional analyses: (1) We tested for potential correlations between the P2 and N400 mean amplitudes in the three experimental conditions. If P2 mean amplitude was functionally linked to N400 modulation, we could expect P2 and N400 mean amplitudes to be correlated. However, this was not the case in any of the conditions (all *p*s > 0.10). (2) We compared the magnitude of the differences (“semantic violation—correct sentence” and “WK violation—correct sentence”; normalized values) in the P2 and N400 time-windows using profile analyses. The results revealed a significant time-window effect [*F*_(1,34)_ = 21.72, *p* < 0.001] and a significant difference effect [*F*_(1,34)_ = 26.96, *p* < 0.001] showing that the magnitude of the N400 effect was larger than the P2 effect, and that the semantic violation effect was larger than the WK violation effect. The time-window × difference interaction was also marginally significant [*F*_(1,34)_ = 4.15, *p* = 0.05] showing that the increase in effect magnitude from P2 to N400 was larger for semantic (*p* < 0.001) than WK (*p* < 0.01) violations. This profile analysis further supported the idea that P2 effects cannot simply be accounted for by N400 effects and vice-versa, since effect magnitudes increased significantly between time-windows. (3) We performed another ANOVA comparing SV, DK (“Don't know”) and correct sentences (see Figure 4 for ERP waves and Figure 5 for mean amplitude values). We did so because visual inspection of the ERP data suggested that P2 and N400 components were modulated differently in SV and DK conditions, suggesting that P2 effects were not byproducts of N400 modulations. The ANOVA performed on P2 mean amplitudes revealed a significant effect of region and a significant condition × region interaction (see Table 4A for statistical results). There was no other significant effect or interaction. *Post-hoc* analysis of condition x region interaction (Bonferroni test; see Table 4B) showed that P2 differences were due to correct sentences eliciting larger P2 amplitudes as compared to the other two conditions (which were not significantly different from one another), over the frontal region. However, the three conditions did not significantly differ over the central and parietal regions. Thus, P2 mean amplitude was sensitive to semantic violations and to an inability to check semantic plausibility (because of a lack of knowledge).

**Table 4A:**
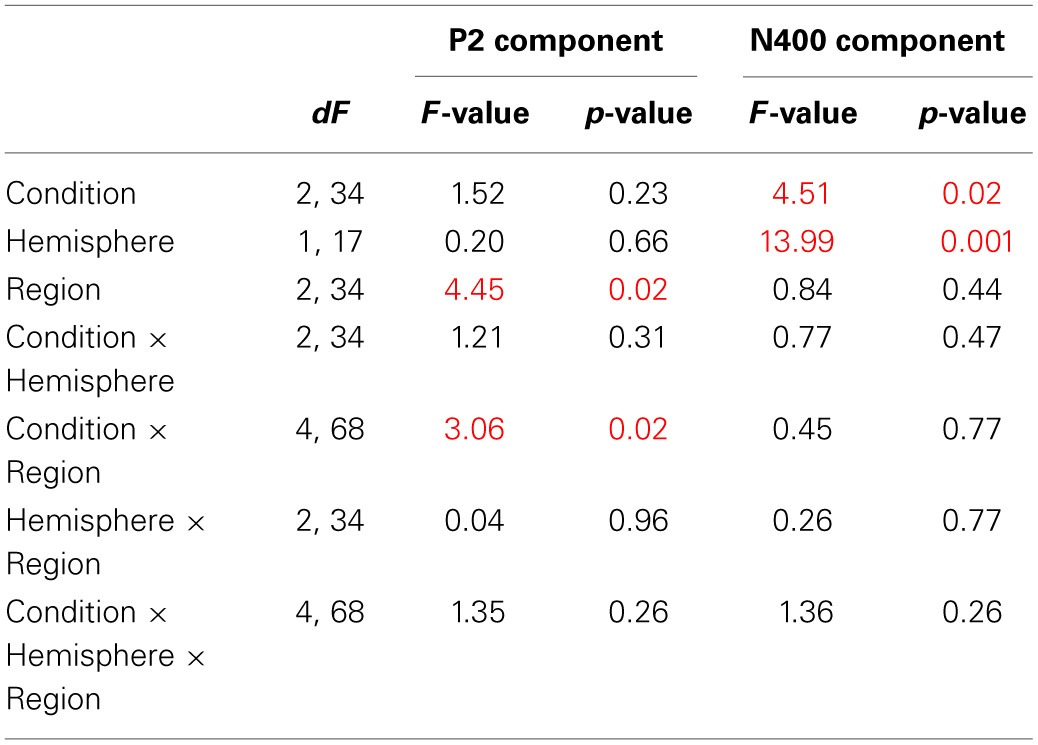
**General ANOVA for CS vs. DK vs. SV comparison**.

CS, correct sentences; DK, don't know sentences; SV, semantic violations; dF, degree of freedom; Significant effects and interactions are labeled in red.

**Table 4B:**
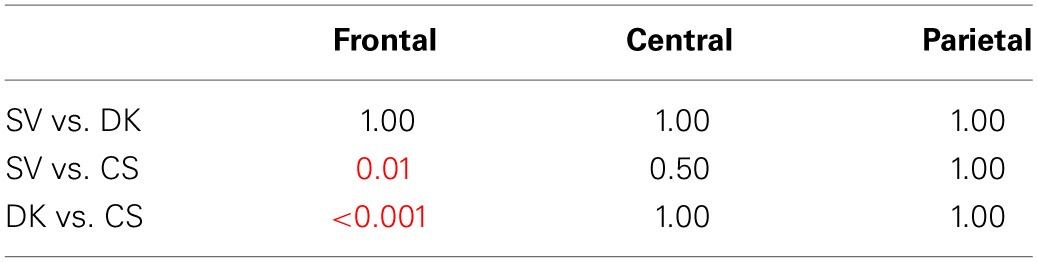
**P2 *Post-hoc* analysis—Bonferroni test of the condition × region interaction**.

The overall ANOVA on N400 mean amplitude showed significant effects of condition and hemisphere (see Table 4A). There was no effect of region and none of the interactions were significant. *Post-hoc* analysis of the condition effect (Bonferroni test; see Table 4C) revealed that N400 mean amplitude was larger for SV than correct sentences. DK sentences did not differed from SV ones. Most importantly, DK and correct sentences did not differ significantly in the N400 time-window (see Figures 4, 5). Thus, N400 mean amplitude was sensitive to semantic violations but not the inability to check semantic plausibility.

**Table 4C:**
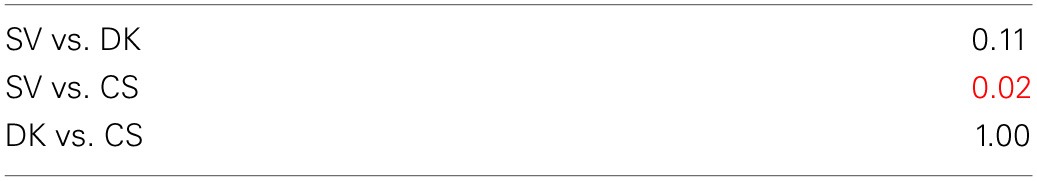
**N400 *Post-hoc* analysis—Bonferroni test of the condition effect**.

## Discussion

The goal of the present study was to investigate whether readers retrieve and integrate literal semantic and world knowledge information simultaneously or in sequence during sentence comprehension. To reduce potential confounding effects of anticipation in the N400 modulations, we used sentences with low constraint contexts. Furthermore, we maximized the ecological validity of our ERP results by distinguishing true and false sentences based on individual knowledge. Furthermore, we investigated not only the N400 but also the P2 component modulation elicited by the critical word of sentences in three conditions: (1) correct sentences (true sentences); (2) sentences with semantic violations (impossible sentences); (3) sentences with world knowledge violations (false sentences).

Two main results were observed. First, semantic violations, and world knowledge violations elicited a larger N400 component as compared to correct sentences. This result replicates previous observations by Hagoort et al. (2004). Second, and more importantly, sentences with semantic violations significantly differed from both world knowledge violations and correct sentences in the P2 time-window. This latter result is perhaps the most relevant contribution of the present study, since it reveals that semantic and world knowledge violations seem to be processed with different time-courses.

### Latency differences between semantic and world knowledge integration

Semantic and world knowledge violations have been shown previously to elicit a larger N400 component as compared to control sentences (Hagoort et al., 2004). This observation suggests that, at some point in time, both types of information are concurrently processed. Here, however, differences between semantic violations and correct sentences appeared before world knowledge violations had any effect (in the P2 range; see Landi and Perfetti, 2007; Penolazzi et al., 2007; Pinheiro et al., 2010). To the extent that semantic and world knowledge violations reveal the time at which the brain integrates information about the specific meaning of words and their truth-value, we can conclude that speakers integrate literal meaning before sentential truth value rather than simultaneously. Note that we interpret our results in relation to the “classical” semantic integration account of the N400. We choose this framework in order to compare our results with those obtained by Hagoort et al. (2004). Other interpretational frameworks could have been chosen, such as the long-term memory access account (see for instance Kutas and Federmeier, 2011). Since the theoretical explanation of the N400 is beyond the scope of this study, we do not discuss this issue further and merely argue that our data support a two-stage process, sensitive sequentially to literal meaning and then to veracity. Thus, we do not make claims as regards the nature of the process at work, be it integration or long-term memory access.

Although at first glance this conclusion seems at odds with that of Hagoort et al. (2004), according to whom both types of information are integrated simultaneously, we believe it is complementary rather than contradictory. In fact, our results are not necessarily inconsistent with Hagoort et al.'s results regarding the presence of earlier ERP modulations by semantic violations since they focussed their study on N400 modulations and did not report potential differences between conditions in earlier time windows (see also Hald et al., 2006, 2007)^3^. More importantly, in the paper by Hagoort et al. (2004), the cloze probability for critical words in the correct sentences was 49% [range 0–100%; values reported in Hald et al. (2006)]. Sentences in which the critical words cloze probability was 100% might have confounded semantic integration and expectation. When sentences are highly constrained, one specific lexical item is expected, and any word violating this expectancy will likely elicit a large N400 (making the distinction between semantic and world knowledge violation undetectable, as in both cases the critical word violates the expectancy). Note also that previous studies showing an early contextual integration influence revealed that such influence is highly dependent on stimulus variance and probability of occurrence (Sereno and Rayner, 2003; Penolazzi et al., 2007). For instance, Penolazzi et al. (2007) showed that the early P2 semantic effect was modulated by the probability of word occurrence in a given context. Thus, we argue that the lack of early semantic effect in Hagoort et al. (2004)' study might be explained by a large range of critical word cloze probability values.

### Semantic violation effect in the P2 time-window

From a methodological point of view, the semantic violation effect in the P2 time-window could be a by-product of the following N400 semantic effect. Some researchers who observed modulation of the P2 by semantic congruency have suggested that such early semantic effect might be functionally related to later N400 modulation (i.e., due to the onset of the following N400 component; Coulson et al., 2005). In the present study, this interpretation is unlikely given that the topography of the P2 and N400 effects were somewhat different (the P200 was more frontally distributed than the N400; see Landi and Perfetti, 2007 for similar argument for two separate processes). The absence of correlation between P2 and N400 mean amplitude in any of the three conditions and the main effect of time-window in the profile analysis also make this interpretation unlikely. More importantly, the DK condition elicited P2 mean amplitude similar to that elicited in the SV condition despite the absence of any subsequent modulation in the N400 window. Assuming that the reduction in P2 mean amplitude in SV and DK sentences (compared to correct sentences) reflects the same cognitive process, it is most probably not a by-product of the subsequent N400 effect.

ERP results in the DK condition also provide interesting clues for the theoretical interpretation of both P2 and N400 effects. We cannot draw definitive conclusions from the present data because the DK condition only concerned 10% of the trials. Nevertheless, it seems that P2 is sensitive to semantic violations and to participants' inability to check semantic plausibility, but not sensitive to veracity (as long as content can be interpreted). By contrast, the N400 component appears sensitive to both literal meaning and veracity. Thus, we argue that the N400 reflects simultaneous integration of word meaning, paralinguistic information, and information stored in long-term memory. This interpretation is compatible with Hagoort et al. (2004)'s conclusions, but also with other studies having suggested that word meaning is concurrently processed with indexical properties of speech, social aspects of language, gestures, etc. in the N400 window (see for instance Kelly et al., 2004; van Berkum et al., 2008; van den Brink et al., 2012). Nevertheless, our results show that earlier in time, the brain makes a difference between information that is semantically interpretable or contextually meaningless (see Baccino and Manunta, 2005), before world knowledge stored in long-term memory is taken into account. This early effect of semantic processing modulating the P2 component is consistent with several previous studies (see Baccino and Manunta, 2005; Landi and Perfetti, 2007; Penolazzi et al., 2007; Wirth et al., 2008; Pinheiro et al., 2010). Even if the P2 component is classically thought to reflect processes related to higher order visual feature detection and analysis (Hillyard and Münte, 1984; Luck and Hillyard, 1994; Federmeier and Kutas, 2002; Federmeier et al., 2005), several studies have now reported P2 effects in several aspects of language processing such as lexical-semantic violations. Our results provide new evidence for early semantic access and contextual integration during sentence processing, around 200–250 ms after stimulus onset (Martín-Loeches et al., 2004; Landi and Perfetti, 2007; Penolazzi et al., 2007; Pinheiro et al., 2010; Regel et al., 2010; see also Pulvermüller, 2001; Pulvermüller et al., 2001, 2009; Barber and Kutas, 2007). The present results are also consistent with previous observations of early cross-modal semantic integration: Studies of gesture-speech integration showed that semantically congruent and semantically incongruent gesture-speech combinations start to differ in the P2 time-window (see for instance Kelly et al., 2004, 2009).

### Potential effects of lexical-semantic relationships

The observation of a reduced N400 mean amplitude in world knowledge violations relative to semantic violations could be boiled down to lexical-semantic priming between the critical word and previous words in the sentence context (see Federmeier and Kutas, 1999a,b; Federmeier et al., 2002). Given the way in which semantic and world knowledge violations were constructed, semantic violations could be considered between-category violations (outside the semantic field of the sentence context; e.g., “They wanted to make the hotel look more like a tropical resort. So, along the driveway, they planted rows of *tulips*”—*palms* being the expected exemplar; Federmeier and Kutas, 1999b; Federmeier et al., 2002) and world knowledge violations could be perceived as within-category violations (within the semantic field of the sentence context; e.g., “… So, along the driveway, they planted rows of *pines*”). Several studies have shown that the N400 effect was smaller for within-category as compared to between-category violations, because of the organization of long-term semantic memory (Federmeier and Kutas, 1999a,b; Federmeier et al., 2002). Thus, the similar pattern of N400 reduction observed here could be explained in terms of mere lexical-semantic priming rather than a difference between veracity and plausibility verification. In other words, the decrease of the N400 effect in the WK violation condition (relative to the semantic violation condition) may not be explained by the fact that participants had to integrate critical words against knowledge stored in long-term memory, but rather by the semantic relatedness of the critical words with other words in the sentence. According to the theoretical framework within which we choose to define the two types of violations (cf. Introduction; see also Hagoort et al., 2004; Hald et al., 2006, 2007 for similar definitions), world knowledge violations are within-category violations and semantic violations are between-category violations. Thus, we acknowledge that there might not be any specific cognitive process dedicated to integrating words against knowledge stored in long-term memory, but rather a common and broad processing system for semantic integration driven by the degree of mismatch between the meaning of a word and that elicited by the preceding context. Our results cannot shed light onto this alternative. Nevertheless, it is likely that cognitive operations beyond lexical-semantic integration are at work within the early time-window of the P2 and that semantic evaluation does not proceed all at once for the two scenarios tested here.

We would like to raise a potential limitation of the present study, being that eye movements may have influenced to some extent ERP effects observed in the present study. In fact, previous studies have suggested that eye movements may differ for normal and violated sentence comprehension (see Clifton et al., 2007; Liversedge et al., 2011). Out of the scope of the present study, further research should focus on differentiating how much violation effects arise from eye vs. brain activity, separation of signals generated by the eyes and the brain being always challenging. Nevertheless, we are confident regarding the validity of our conclusions given that all analyses have been run with eye blink trials removed and that the results were essentially the same despite the drop in statistical power.

## Conclusion

To conclude, the present study showed that some aspect(s) of semantic and world knowledge violations are processed with different time-courses. Readers access literal semantic information ~200 ms before they access factual knowledge about the world. Consistent with previous results, we observed the first significant effects of semantic violations around 200 ms after the critical word onset. Then, further down the line, in the vicinity of the N400, both types of information are processed concurrently.

### Conflict of interest statement

The authors declare that the research was conducted in the absence of any commercial or financial relationships that could be construed as a potential conflict of interest.

## Footnotes

^1^The post-test revealed that 20% of true sentences were rated as false or “unknown” by the participants, and 27% of false sentences were rated as true or “unknown.”

^2^Cloze probability of a word in a particular sentence is defined as the percentage of time it is produced by a group of control participants asked to complete the sentence.

^3^Hagoort and colleagues reported time-course analyses in supporting online material, which revealed no differences between world knowledge violations and semantic violations in the P2 time-window. The two conditions started to diverge around 480 ms post-stimulus onset (in the N400 time-window). Nevertheless, they did not report time-course analyses comparing semantic violations vs. correct sentences and world knowledge violations vs. correct sentences.
